# The Technique of Halsted's Operation for Cancer of the Breast
1Read before the Plymouth Medical Society, October 27th, 1900.


**Published:** 1900-12

**Authors:** C. Hamilton Whiteford

**Affiliations:** Medical Officer to the Provident Branch of the Plymouth Public Dispensary


### THE TECHNIQUE OF HALSTED'S OPERATION FOR CANCER OF THE BREAST.[1]

BY

C. Hamilton Whiteford, M.R.C.S., L.R.C.P.,

*Medical Officer to the Provident Branch of the Plymouth Public Dispensary.*

---

Halsted's account of this operation, as given by Butlin in the *British Medical Journal* of December 3rd, 1898, has always appeared to me capable of improvement in several details. Several of these I employed in the following case :

A married nullipara, aged 31, was sent to me for operation on August 14th last by Dr. Clay. She had a hard irregular mass, the size of a walnut, in the upper and outer segment of the left breast, movable on the chest-wall and not involving the skin. No glands palpable. Prior to removal I incised the tumour, which proved to be a scirrhus. I followed Halsted's description, with the following modifications:—The primary incision was made through skin only. No advantage is gained by cutting through the subcutaneous fat; and

[1] Read before the Plymouth Medical Society, October 27th, 1900.

if this fat is already involved by the growth, incising it may possibly disseminate cancer cells. The lesser pectoral instead of being divided at its centre was detached from the coracoid process, turned down and removed with the greater pectoral and breast. The clavicular portion of the greater pectoral was divided at its origin from the clavicle. The actual operation took three hours and three-quarters; the patient stood it well. Three pints of hot saline solution, with half ounce of brandy, were injected with a Higginson's syringe into the right median cephalic vein at the end of the operation. The patient was under chloroform four hours and a quarter, had no after-sickness, and felt hungry the same evening. The lower edges of the wound, in spite of very free undermining, could not be approximated, and two inches of the apex of the axillary flap sloughed. Except for this the patient did well.

Mr. Butlin, who is the chief advocate in England of this operation, has very kindly given me the results of his large experience. He says: "I have performed the operation probably more than a hundred times, and am very well satisfied with it. Sloughing of a small portion of the axillary flap is not uncommon. I believe the best way to guard against it is not to make the flap too large, but to cut the lower flap rather high up along the anterior pectoral line. I have not yet discovered whether the leaving of fat in the flaps prevents sloughing, but I do not think it does. I do not remove the lesser pectoral muscle, unless there is disease of glands almost adherent to it. I never bring the cut portions together, because there is absolutely no advantage in doing so, and there is great discomfort produced by bringing together cut muscle."

In future I shall adopt Mr. Butlin's suggestion in cutting the lower flap. I make it a rule to incise every breast tumour prior to removal, and, if it turns out to be malignant, stitch up the exploratory incision and discard all instruments already used. By so doing the risk of disseminating cancer is rendered infinitesimal.

I recently operated on a widow, aged 40, with a hard irregular mass, the size of a pigeon's egg, deep in the right breast, one inch above the nipple. I had no doubt but that it was malignant, and two other medical men agreed with me. On incision the mass was seen to consist of three adenomata grouped together. I have also seen within the last few days a young woman, with a history of mastitis and abscess seven years previously, who had an irregular thickening of the upper and outer portion of the right breast, which had recently become somewhat tender, but had if anything decreased in size. The unanimous diagnosis of those who saw it was inflammatory thickening left by the old mastitis. On incision the thickening proved to be a scirrhus.

The amount of blood lost by working from above downwards is, as pointed out by Halsted, very small, because the vessels are freely exposed and ligatured close to the main trunks once and for all, instead of the same vessels being repeatedly divided and clamped, which is what usually happens when clearing the axilla from below. I attach the greatest importance to having the patient on a hot-water bed during the operation, which is of necessity a severe one, and to intravenous injection of saline solution at the finish. At least five scalpels are required to do the operation comfortably. Halsted spends between three and four hours on the operation, which he finds very fatiguing. No operation has ever tired me so much, although sitting down most of the time.

